# Henoch-Schönlein Purpura in a child induced by Valproic Acid

**DOI:** 10.1186/2045-7022-5-S1-P15

**Published:** 2015-03-11

**Authors:** Dimitra Koumaki, Evangelia Papadavid, Ioannis Panayiotides, Ekaterina Shtukkert, Argyrios Dinopoulos, Dimitrios Rigopoulos

**Affiliations:** 1 University of Athens Medical School, "Attikon" University Hospital, 2nd Department of Dermatology and Venereology, Haidari, Athens; Greece; 2 University of Athens Medical School, 2nd Department of Pathology, Haidari, Athens; Greece; 3 Medical School University of Athens,“Attikon” General Hospital, 3rd Department of Paediatrics, Haidari, Athens, Greece

## Background

Here we report a case of a child who developed Henoch–Schönlein purpura (HSP) after administration of valproic acid.

## Case report

A 14-year-old male was admitted with a five months history of recurrent purpuric papules on his buttocks and lower limbs lasting up to four days with a burning sensation. The patient had a 10 year medical history of epileptic absences treated with valproic acid 300 mg daily before the initiation of purpura. Laboratory screening for cutaneous vasculitis was unremarkable including full blood count with differential, liver function tests, renal and coagulation profile. Results of testing for celiac and thyroid antibodies were negative . Immunological testing for anti-DNA antibodies, IgG, IgA, IgE, IgM, plasma levels of C3 and C4 factors were also negative. Plasma levels of valproic acid were 27,2 μg/ ml (therapeutic serum concentrations range from 50 to 100μg/ml). A skin biopsy showed a massive inflammatory infiltrate in the dermis extravasation of neutrophils and erythrocytes and deposition of fibrin. Direct immunofluorescence demonstrated deposits of IgA in the vascular wall that confirmed the diagnosis of Henoch–Schönlein purpura. On discontinuation of valproate, the child was fully recovered from purpura seven days after the withdrawal of the drug. The child was challenged via hyperventilation for seizures with negative results. He was followed up for more than one year with no recurrence of the rash or epilepsy.

## Conclusion

This is the first reported case of Henoch–Schönlein purpura induced by valproic acid. Physicians should be aware of the range of severity of Henoch–Schönlein purpura and discontinue the drug Sodium valproate should be added to the list of drugs that may cause Henoch–Schönlein purpura.

## Consent

Written informed consent was obtained from the patient for publication of this abstract and any accompanying images. A copy of the written consent is available for review by the Editor of this journal.

